# Clinical study of Edaravone Dexborneol combined with Tirofiban in treatment of Acute Cerebral Infarction

**DOI:** 10.12669/pjms.40.1.7630

**Published:** 2024

**Authors:** Yuxia Mi, Zhigang Hou, Jiaqi Liu, Caixia Zhang

**Affiliations:** 1 Yuxia Mi, Department of Neurology, Cangzhou People’s Hospital, Cangzhou 061000, Hebei, P.R. China; 2 Zhigang Hou, Department of Neurology, Cangzhou People’s Hospital, Cangzhou 061000, Hebei, P.R. China; 3 Jiaqi Liu, Department of Neurology, Cangzhou People’s Hospital, Cangzhou 061000, Hebei, P.R. China; 4 Caixia Zhang, Department of Neurology, Cangzhou People’s Hospital, Cangzhou 061000, Hebei, P.R. China

**Keywords:** Acute cerebral infarction, Edaravone dexborneol, Tirofiban, Inflammatory factor, Hemodynamic index

## Abstract

**Objective:**

To explore the clinical efficacy and safety of edaravone dexborneol combined with tirofiban in the treatment of acute cerebral infarction.

**Methods:**

This is a retrospective study. A total of 80 patients with acute cerebral infarction (ACI) treated in Cangzhou People’s Hospital from March 2018 to December 2021 were selected, and randomly divided into the routine group(n=40) and intervention group(n=40) according to the principle of random draw. Neurological deficits in the two groups were evaluated and compared before and after treatment using the National Institutes of Health Stroke Scale (NIHSS). The quality of life of the patients was evaluated by the modified Rankin Scale (mRS).

**Results:**

Before treatment, NIHSS and mRS scores of the two groups were not statistically significant(*p>*0.05); the levels of Vmin and Qmin in the two groups showed no statistical significance(*p>*0.05); no statistical significance was found in CRP or IL-6 levels between the two groups(*p>*0.05). After treatment for 14 days, the NIHSS and mRS scores of the two groups both decreased, which was more significant in the intervention group(*p<*0.05); Vmin and Qmin levels increased in both groups, which was more obvious in the intervention group(*p<*0.05); CRP and IL-6 levels reduced in both groups, which was more remarkable in the intervention group(*p<*0.05). The incidences of relevant adverse drug reactions presented no differences between the two groups during treatment(*p>*0.05).

**Conclusion:**

Edaravone dexborneol combined with tirofiban in the treatment of ACI can effectively improve patients’ neurological deficits, quality of life and cerebral blood flow, and reduce inflammatory factor levels, have significant clinical efficacy and high clinical treatment safety.

## INTRODUCTION

Acute cerebral infarction (ACI) is one of the most common cerebrovascular diseases in the clinic. With the improvement of living standards and the aging of the population, the incidences of chronic diseases such as primary hypertension and Type-2 diabetes have increased, leading to increased clinical incidences of acute cerebrovascular diseases, which have a serious impact on the health and quality of life of patients.^1^ Acute cerebral infarction (ACI) is characterized by rapid onset. Without timely diagnosis and treatment, it can cause limb dysfunction and even death as the occurrence and development of the disease.^2^ Cerebral atherosclerosis is an important pathological basis of acute cerebrovascular diseases. After cerebral atherosclerosis, thrombus shedding or rapid platelet aggregation is an important cause of ACI. After the occurrence of cerebral infarction(CI), the blood supply to corresponding brain tissues is affected, which leads to ischemia and hypoxia of brain tissues and then denaturation and necrosis, thereby causing damage to brain tissues and neurological functions, and directly affecting the body’s limb motor function.^3^ Relaxing blocked cerebral vessels, restoring the blood supply to brain tissues, as well as protecting brain tissues and neurological functions are the main measures for ACI treatment. Tirofiban is a new clinical antiplatelet drug, which can quickly inhibit platelet aggregation, and has important value in dredging blocked vessels and opening the microcirculation.^4^ Edaravone dexborneol is a novel neuroprotective agent in clinical practice. Our objective was to explore the efficacy and safety of edaravone dexborneol combined with tirofiban in patients with ACI.

## METHODS

This is a retrospective study. A total of 80 patients with ACI treated in Cangzhou People’s Hospital from March 2018 to December 2021 were selected for this study.

### Ethical Approval

The study was approved by the Institutional Ethics Committee of Cangzhou People’s Hospital (No.: K2020-162(12.9); date: December 15, 2020), and written informed consent was obtained from all participants.

### Inclusion criteria:

- Patients were admitted due to acute symptoms such as headache, nausea, vomiting and limb dysfunction, and received brain CT or MR examination after admission, which definitely diagnosed ACI;
- Patients presented stable vital signs after emergency treatment;
- Patients had indications for edaravone dexborneol and tirofiban after evaluating their general Condition and disease condition;
- Patients and their families voluntarily participated in this treatment and study after information.

### Exclusion criteria:

- Patients presented stable vital signs after emergency treatment;
- Patients had indications for edaravone dexborneol and tirofiban after evaluating their general condition and disease condition;
- Complicated cerebral hemorrhage or brain tumors;
- Uncontrollable blood pressure;
- Coagulation dysfunction or immune system diseases.

Using the random number method, the patients were randomly divided into the routine group (routine treatment combined with tirofiban, n= 40) and the intervention group (routine treatment combined with tirofiban, edaravone dexborneol, n=40). The general clinical data showed no statistical significance between the two groups(*p>* 0.05), as seen in Table-I.

**Table-I:** Clinical data.

Group	n	Male/female (n)	Age (year)	Time from onset to admission (h)	Complicated basic diseases (n)	

					Primary hypertension	Diabetes
Routine group	40	23/17	62.5±8.7	12.2±3.2	19	15
Intervention group	40	25/15	61.9±8.9	12.1±2.9	21	17
*χ*^2^/t	-	0.208	0.304	0.146	0.200	0.208
*p*	-	0.648	0.761	0.883	0.654	0.648

The patients in the routine group received routine treatment. After admission, thrombolytic therapy with alteplase was given to patients with indications for thrombolytic therapy, and interventional therapy to those with indications for interventional therapy. In addition, the patients’ blood pressure, blood glucose and blood lipid levels were actively controlled stable, symptomatic treatment was performed for improving cerebral metabolism, cerebral circulation and cerebral protection, and nutritional support was strengthened. Aspirin Enteric-Coated Tablets (Bayer Healthcare Co., Ltd., approval No.: 2011022106) were orally administrated once a day, 100 mg/time. Atorvastatin Calcium Tablets (Pfizer Pharmaceuticals Co. Ltd., approval No.: 16214542) were orally administrated once a day, 20 mg/time. According to body weight, tirofiban (Nankai Yungong Pharmaceutical Co., Ltd., approval No.: 221521401) was intravenously dripped at 0.4 ug/kg.min for 30 min, followed by continuous intravenous infusion at 0.1 ug/kg.min for 48 h. Based on the routine group, the intervention group was additionally treated with an intravenous drip of 37.5 mg edaravone dexborneol (Nanjing Simcere Dongyuan Pharmaceutical Co., Ltd., approval No.: H20200007) in 100 mg 0.9% sodium chloride injection, two times/d, for consecutive 14 days.

### Observation indexes

Before treatment and 14 days after treatment, the neurological deficits of the patients were scored using the National Institutes of Health Stroke Scale (NIHSS), including 15 indexes such as limb movement, visual field, gaze function and cognitive function, with a total score of 42. The higher the score, the severer the neurological deficits.^5^ Before treatment and 14 days after treatment, the quality of life of the patients was evaluated by the modified Rankin Scale (mRS) as grade 0-6: 0, no symptom; 1, mild symptoms, not affecting the normal life and work of patients; two, mild dysfunction, presenting inability to complete previous work well, but no need for others’ help; three, moderate dysfunction, with walking needing no help, but daily life needing some help; four, severe dysfunction, with both walking and daily life needing others’ help; five, extremely severe dysfunction, manifested as fecal and urinary incontinence and bed-ridden; six, death.^6^ Before treatment and 14 days after treatment, the middle cerebral artery was detected, and the minimum blood flow velocity (Vmin) and minimum blood flow (Qmin) were measured in the two groups using transcranial color Doppler ultrasound. Before treatment and 14 d after treatment, five ml of elbow venous blood was collected into anticoagulant tubes under fasting condition in the morning, and the levels of C-reactive protein (CRP) and interleukin-6 (IL-6) were determined and compared between the two groups. During 14-d treatment, the incidences of rashes, hepatic dysfunction, dizziness and headache, and gastrointestinal discomfort were counted and compared between the two groups.

### Statistical Analysis

The obtained data were analyzed using SPSS 24.0 statistical software package. Age, time from onset to admission, neurological deficit and quality of life scores, hemodynamic indexes, and inflammatory factor levels conformed to the normal distribution, and were expressed as (*χ̅* ± *s*) and analyzed by the *t* test. Gender, complicated basic diseases and adverse drug reactions were expressed as rate and analyzed with the *χ*^2^ test. The power of test / confidence interval was 95%. *α*= 0.05 was considered statistically significant.

## RESULTS

Before treatment, NIHSS and mRS scores of the two groups were not statistically significant (*p>* 0.05). After treatment for 14 days, the NIHSS and mRS scores of the two groups both decreased compared with those before treatment, which was more significant in the intervention group (*p<* 0.05). Table-II.

**Table-II:** Comparison of neurological deficits and quality of life between the two groups (score, *χ̅*±*s*).

Group	n	NIHSS score		mRS score	

		Before treatment	14 d after treatment	Before treatment	14 d after treatment
Routine group	40	28.3±5.1	19.8±3.1*	4.8±0.5	3.1±0.5*
Intervention group	40	28.1±5.6	15.5±2.6*	4.7±0.9	2.2±0.3*
*t*	-	0.167	6.721	0.614	9.761
*p*	-	0.867	0.000	0.540	0.000

***Notes:***

* compared with before treatment, *p<* 0.05.

Before treatment, the levels of Vmin and Qmin in the two groups showed no statistical significance (*p>* 0.05). After treatment for 14 days, Vmin and Qmin levels increased in both groups compared with those before treatment, which was more obvious in the intervention group (*p<* 0.05). Table-III.

**Table-III:** Comparison of cerebral hemodynamic indexes between the two groups (cm/s, *χ̅*±*s*).

Group	n	Vmin		Qmin	

		Before treatment	14 d after treatment	Before treatment	14 d after treatment
Routine group	40	7.1±1.1	8.9±1.2*	3.9±0.6	4.8±0.8*
Intervention group	40	7.2±1.3	10.3±1.5*	3.8±0.8	5.3±0.7*
*t*	-	0.371	4.609	0.632	2.974
*p*	-	0.711	0.000	0.528	0.004

***Notes:***

* compared with before treatment, *p<* 0.05.

Before treatment, no statistical significance was found in CRP or IL-6 levels between the two groups (*p>* 0.05). After treatment for 14 days, CRP and IL-6 levels reduced in both groups compared with those before treatment, which was more remarkable in the intervention group (*p<* 0.05). Table-IV. The incidences of relevant adverse drug reactions presented no differences between the two groups during treatment (*p>* 0.05). Table-V.

**Table-IV:** Comparison of inflammatory factor levels between the two groups (*χ̅*±*s*).

Group	n	CRP(mg/L)		IL-(pg/mL)	

		Before treatment	14 d after treatment	Before treatment	14 d after treatment
Routine group	40	32.2±6.5	18.7±3.6*	16.4±2.6	8.7±1.9*
Intervention group	40	31.9±7.4	11.4±2.5*	16.1±2.8	5.3±1.3*
*t*	-	0.192	10.533	0.496	9.340
*p*	-	0.847	0.000	0.620	0.000

***Notes:***

* compared with before treatment, *p<* 0.05.

**Table-V:** Comparison of adverse drug reactions between the two groups [n (%)].

Group	n	Rashes	Hepatic dysfunction	Dizziness and headache	Gastrointestinal discomfort
Routine group	40	0(0.00)	1(2.50)	2(5.00)	2(5.00)
Intervention group	40	1(2.50)	2(5.00)	2(5.00)	1(2.50)
*χ* ^2^	-	1.012	0.346	0.000	0.346
*P*	-	0.314	0.556	1.000	0.556

## DISCUSSION

Edaravone dexborneol is a new neuroprotective drug in clinical practice, which can protect brain tissues and nerves by clearing inflammatory factors and oxygen free radicals in brain tissues.^7^,^8^ In the present study, edaravone dexborneol combined with tirofiban was given to patients with ACI, which significantly reduced the neurological damage and improved the quality of life (*p<* 0.05). The degree of neurological deficits is an important standard to reflect the severity of ACI in patients. The larger the area of brain tissue damage, the severer the corresponding neurological deficits and the lower the quality of life.^9^ Edaravone dexborneol can help reduce brain edema and secondary damage to brain tissues and nerves caused by inflammatory factors by clearing oxygen free radicals and inflammatory factors in brain tissues, thus improving the efficacy of brain tissue and nerve protection.^10^ Our results showed that edaravone dexborneol combined with tirofiban in the treatment of ACI could improve the cerebral arterial hemodynamic indexes of patients (*p<* 0.05). After CI, the corresponding cerebral arteries are blocked, affecting the blood supply to brain tissues. Detection of cerebral arterial hemodynamic indexes can reflect the blockage and opening in brain tissues. Based on tirofiban effectively inhibiting platelet aggregation, edaravone dexborneol can assist to clear inflammatory factors in brain tissues, thus reducing cerebrovascular injury, and then improving cerebral arterial hemodynamics. Some scholars have given edaravone dexborneol to assist in the treatment of patients with ACI, which effectively reduces inflammatory factor levels and improves the hemodynamic indexes of cerebral arteries.^11^,^12^

As one of the common cerebrovascular diseases in the clinic, ACI is an acute occlusive cerebrovascular disease with a variety of causes. With the improvement in clinical diagnosis and treatment technology, the clinical mortality of patients with ACI has decreased, but cerebral vascular occlusion after CI can cause damage to corresponding brain tissues and nerves, resulting in dysphagia, limb dysfunction, etc., which seriously affect the quality of life of patients.^13^,^14^ In addition, many clinical treatment methods for ACI have emerged. While maintaining the vital signs of patients, strengthening brain tissue protection and reducing the degree of neurological damage can effectively relieve neurological damage, reduce dysfunction and improve the quality of life of patients after CI.^15^,^16^ Platelet aggregation is an important cause of atherosclerosis and thrombosis, and also an important pathological basis for ACI. Inhibition of platelet aggregation is an important measure for the clinical treatment of ACI. It has been pointed out that the application of platelet aggregation inhibition in patients with ACI can effectively inhibit platelet aggregation in blocked cerebral arteries, rapidly restore the blood supply to brain tissues, and reduce neurological damage.^17^ Tirofiban is a novel antiplatelet aggregation drug in clinical practice, which can inhibit platelet aggregation by inhibiting platelet membrane glycoprotein IIb/IIIa receptor.^18^ Some scholars have pointed out that tirofiban can effectively inhibit platelet aggregation, improve cerebral microcirculation and promote the opening of cerebral collateral circulation in patients with ACI.^19^

The results of our study also showed that the combination of edaravone dexborneol and tirofiban in the treatment of ACI could significantly reduce the levels of inflammatory factors in patients (*p<* 0.05). After CI, brain tissue denaturation and necrosis occur, followed by brain edema, inflammatory factor release and oxidation reaction, further leading to secondary damage to brain tissues and nerves, which is an important cause for aggravating patients’ disease.^20^ daravone dexborneol can protect brain tissues and nerves mainly through clearing inflammatory factors and oxygen free radicals in brain tissues, thereby reducing the levels of inflammatory factors in patients. In our study, no differences were found in the incidences of relevant adverse drug reactions between the two groups during treatment (*p>* 0.05), which confirms that the combination of edaravone dexborneol and tirofiban has high safety in the treatment of ACI.

### Limitations of the study

It includes fewer cases and short follow-up time, no other treatment options are included for comparative analysis with this study. In response to this, further intervention trials are needed in the future to confirm these results.

## CONCLUSIONS

Edaravone dexborneol combined with tirofiban in the treatment of ACI can effectively improve patients’ neurological deficits, quality of life and cerebral blood flow, and reduce inflammatory factor levels, with significant clinical efficacy and high clinical safety.

### Authors’ Contributions:

**YM and ZH** carried out the studies, participated in collecting data, are responsible and accountable for the accuracy and integrity of the work.

**JL** performed the statistical analysis and participated in its design.

**CZ** participated in acquisition, analysis the manuscript.

All authors read and approved the final manuscript.
